# Tumor-Infiltrating T Cells Can Be Expanded Successfully from Primary Uveal Melanoma after Separation from Their Tumor Environment

**DOI:** 10.1016/j.xops.2022.100132

**Published:** 2022-03-01

**Authors:** Gülçin Gezgin, Marten Visser, Dina Ruano, Saskia J. Santegoets, Noel F.C.C. de Miranda, Pieter A. van der Velden, Gregorius P.M. Luyten, Sjoerd H. van der Burg, Els M. Verdegaal, Martine J. Jager

**Affiliations:** 1 Department of Ophthalmology, Leiden University Medical Center, Leiden, The Netherlands; 2 Department of Medical Oncology, Oncode Institute, Leiden University Medical Center, Leiden, The Netherlands; 3 Department of Pathology, Leiden University Medical Center, Leiden, The Netherlands

**Keywords:** Immunotherapy, Tumor-infiltrating lymphocytes, Tumor-infiltrating T cells, Uveal melanoma, BAP1, BRCA1-associated protein 1, CTLA4, cytotoxic T-lymphocyte-associated protein 4, IFN-γ, interferon γ, IL, interleukin, *PD-1*, *programmed cell death protein 1*, TILs, tumor-infiltrating lymphocytes, TIM-3, T-cell immunoglobulin and mucin-domain containing 3, TME, tumor microenvironment, UM, uveal melanoma

## Abstract

**Purpose:**

To evaluate whether expanded tumor-infiltrating lymphocytes (TILs) can be obtained from primary uveal melanoma (UM) for potential use as adjuvant treatment in patients at risk of developing metastatic disease.

**Design:**

Experimental research study.

**Participants:**

Freshly obtained primary UM from 30 patients.

**Methods:**

Three different methods were used to expand TILs: (1) direct culture from small fragments of fresh tumor tissue, (2) single-cell tissue preparation by enzymatic digestion and subsequent enrichment of mononuclear cells, and (3) selection of CD3^+^ T cells using magnetic beads. Surface expression of costimulatory and inhibitory T-cell markers and T-cell reactivity against autologous tumor cells was assessed. Clinical, histopathologic, genetic, and immunologic characteristics of the tumors were compared with the capacity to expand TILs and with their reactivity against autologous tumor cells.

**Main Outcome Measures:**

The feasibility of expanding TILs from primary UM, testing their reactivity to autologous UM cells, and evaluating the impact of an immunomodulatory environment.

**Results:**

Direct culture of tumor parts led to successful TIL culture in 4 of 22 tumors (18%), enrichment of mononuclear cells gave rise to TILs in 5 of 12 tumors (42%), while preselection of CD3^+^ T cells with magnetic beads resulted in TIL expansion in 17 of 25 tumors (68%). In 8 of 17 tumors (47%), the TIL cultures comprised UM-reactive T cells. The presence of UM-reactive T cells among TILs was not related to clinical, histologic, genetic, or immunological tumor characteristics. Interestingly, RNA-Seq analysis showed that approximately half of the UM tumors displayed an increased expression of immunomodulatory molecules related to T-cell suppression, such as *galectin 3*, *programmed death-ligand 1*, *cytotoxic T-lymphocyte-associated protein 4*, *indoleamine 2,3-dioxygenase 1*, and *lymphocyte activating 3*, potentially explaining why T cells require optimal removal of tumor components for expansion.

**Conclusions:**

The need to separate TILs from their tumor microenvironment for their successful expansion and the presence of UM-reactive T cells among TILs suggests that these UM-reactive T cells are strongly suppressed in vivo and that UM is immunogenic. These findings indicate that adoptive TIL therapy could be an option as an adjuvant treatment in primary UM patients at high risk of developing metastatic disease.

Uveal melanoma (UM) is the most common primary intraocular tumor in White adults; it is highly malignant and leads to metastatic disease in up to 50% of patients.^1^^,^^2^ Several eye-saving treatments, such as plaque brachytherapy, proton beam therapy, and stereotactic radiotherapy, are options for relatively small primary UM to achieve local tumor control, while enucleation is the treatment option for larger tumors. A significant number of patients eventually demonstrate metastasis in the first 5 years after enucleation, which is correlated with specific tumor characteristics (e.g., large tumor diameter, extraocular growth, and ciliary body involvement) and chromosomal aberrations (e.g., loss of chromosome 3 and gain of chromosome 8q) and specific mutations.^1^^,^^3^^,^^4^

Although checkpoint inhibitors are often successful as a treatment and improve survival in patients with metastatic cutaneous melanoma,^5^^,^^6^ a lack of clinical effectiveness has been reported for patients with metastatic UM.^7^ Treatment with tumor-infiltrating lymphocytes (TILs) is another therapeutic option and has been reported to result in impressive clinical responses in patients with metastasized cutaneous melanoma.^8^^,^^9^ Thus far, only 1 study has demonstrated that isolation and reinfusion of TILs can result in regression of UM metastases.^10^ These TILs were obtained from liver metastases. Tumor-infiltrating lymphocyte therapy may also be used as an adjuvant treatment in primary UM with a high risk of distant metastases developing. Earlier clinical trials on TIL as adjuvant therapy for primary cutaneous melanoma showed a longer relapse-free survival for patients treated with TILs compared with patients not receiving TIL therapy.^11^

Although we and others previously showed that many primary UM are infiltrated by immune cells, including T cells,^12^, ^13^, ^14^ the intraocular tumor tissue itself is considered an immunosuppressive microenvironment. Although immunohistochemical staining has shown the presence of CD3^+^CD4^+^ T cells and CD3^+^CD8^+^ T cells, this coincides with expression of T-cell and tumor-derived immunosuppressive features.^15^^,^^16^ In particular, T-cell–containing tumors show expression of immunosuppressive molecules, such as programmed death-ligand 1, galectin 3, indoleamine 2,3-dioxygenase-1, and lymphocyte activating 3.^17^, ^18^, ^19^, ^20^ Although most tumors show some degree of immune infiltration, this is particularly pronounced in the most malignant tumors, that is, those that have lost 1 chromosome 3 and lack expression of BRCA1-associated protein 1 (BAP1).^12^^,^^18^^,^^21^ In particular, *BAP1*-negative tumors display an immunosuppressive environment with enhanced expression of several genes, such as *CD38*, *indoleamine 2,3-dioxygenase-1*, and *galectin 3.*^12^^,^^15^^,^^17^^,^^18^

Previously, it was shown that primary cutaneous melanoma cells induce significant T-cell expansion, whereas primary UM cells fail to stimulate T-cell proliferation in mixed-lymphocyte cultures. This T-cell inhibitory capacity was lost when UM cells migrated from the eye to the liver and formed hepatic metastases.^22^ Prior attempts to isolate TILs from primary UM used single-cell populations obtained by digesting tumor tissues, and some of these TILs were able to lyse their autologous tumor cells.^23^ However, these experiments were performed with only a few tumors, and low TIL numbers were obtained.

Since metastatic disease occurs in half of patients with UM after primary treatment, adoptive TIL therapy could be an option as an adjuvant treatment after primary UM treatment, in particular for patients with a high risk of developing metastatic UM. To further explore this option, we evaluated the feasibility of isolating and expanding TILs from freshly obtained primary UM using different techniques, determined their reactivity to autologous UM cells, and evaluated whether the extent of immunosuppression in the original tumor microenvironment (TME) determined success or failure to expand TILs.

Our data show that culturing TILs from freshly obtained primary UM is especially effective following their separation from their immunosuppressive TME and that these TILs have the potential to recognize autologous UM. This indicates that UM is immunogenic and that adoptive TIL therapy can be an option as an adjuvant therapy in a primary therapeutic setting for patients with a high risk of developing metastatic disease.

## Methods

### Study Population

Freshly cut tumor specimens were obtained from 30 patients with primary UM who underwent enucleation at the Leiden University Medical Center (Leiden, The Netherlands), between June 2016 and April 2017. Each tumor sample was processed for TIL culture, histopathologic, and genetic evaluation. Tumor fragments or single-cell suspensions were cryopreserved (detailed description below) and stored in liquid nitrogen until further use. Tumor material was handled according to the Dutch National Ethical Guidelines (Code for Proper Secondary Use of Human Tissue) and the tenets of the Declaration of Helsinki (World Medical Association 2013 declaration of ethical principles for medical research involving human subjects). Material was included in the Leiden Ophthalmic Oncology Biobank (identifier, B14.003/DH/sh). This study was approved by the Medical Ethical Committee of the Leiden University Medical Center (identifier, B20.026) and the need for informed consent was waived, following the regulations laid down for use of patient material according to the Federation of Medical Scientific Societies.

### Tumor-Infiltrating Lymphocyte Culture

Tumor-infiltrating lymphocytes were obtained in 3 different ways: (1) cultured directly from small fragments of fresh tumor tissue, (2) single-cell preparation by enzymatic digestion and subsequent enrichment of mononuclear cells with Ficoll (Leiden University Medical Center pharmacy), and (3) after selection of CD3^+^ T cells with Dynabeads (Invitrogen, Thermo Fisher Scientific). In all protocols, the TILs were expanded in T-cell medium (Iscove’s modified Dulbecco’s medium [Life Technologies]) with 7.5% heat-inactivated human serum (Sanquin), 50 U/ml penicillin and streptomycin, and 4 nM glutamin (Lonza) in 24-well plates. The T-cell medium was supplemented with 1.000 IU/ml recombinant human interleukin (IL)-2 (Aldesleukin; Novartis). Wells were split when the cells at the bottom of the well formed a confluent layer or exceeded a concentration of 1.5 × 10^6^ TIL/ml:

1. Fresh tumor material obtained after enucleation was cut into small fragments (1–2 mm^3^). One to 4 tumor fragments were placed in a total of 2 ml T-cell medium in 24-well plates. Every 2 to 3 days, half of the culture medium was refreshed with T-cell medium containing IL-2.^24^
2. Part of each tumor specimen was used to generate single-cell suspensions. First, the tumor samples were cut into small pieces. Subsequently, a single-cell suspension was generated by enzymatic digestion for 30 minutes at 37° C using a mix of collagenase D with a concentration of 10 mg/ml (Roche) and DNase I with a concentration of 3 mg/ml (Roche), followed by spinning with the gentleMACS dissociation procedure (Miltenyi Biotech Gmbh). Subsequently, the obtained single-cell suspension was used to separate lymphocytes from the tumor cells by using Ficoll in a 50-ml Leucosep tube (Greiner Bio-One). The lymphocytes obtained from the interface were then cultured according to the TIL protocol described above.
3. Part of the single-cell suspension obtained in part 2 was used to count T cells, and these T cells were isolated positively from tumor tissue by using anti-CD3 Dynabeads using the manufacturer’s instructions. The acquired TILs were further cultured according to the TIL protocol described above.

Isolated TILs were cryopreserved in 70% T-cell medium plus 20% fetal calf serum (Bodinco) plus 10% dimethyl sulfoxide (Leiden University Medical Center pharmacy) and were stored in liquid nitrogen until use.

### Immunophenotyping

Cryopreserved TIL samples were thawed, and T-cell and inhibitory markers were assessed by flow cytometry staining as previously described.^25^ We used the following antibodies: CD3-V450 (Becton, Dickinson and Company, BD Biosciences), CD4-PE-CF594 (Becton, Dickinson and Company), CD8-APC-Cy7 (Becton, Dickinson and Company), CD279 programmed cell death protein 1 (PD-1)-PeCy7 (Biolegend), T-cell immunoglobulin and mucin-domain containing-3 (TIM-3)-BV605 (Biolegend), and CD152 cytotoxic T-lymphocyte-associated protein 4 (CTLA4)-Pe-Cy5 (Becton, Dickinson and Company). First, samples were stained with LIVE/DEAD Fixable Yellow Dead Cell Stain Q-dot 585 (Life Technologies) for 20 minutes in the dark at room temperature. After incubation, the cells were washed in phosphate-buffered saline (Fresenia Kabi) and centrifuged. In addition, the cell pellet was resuspended with the antibody mixtures as described above and incubated for 30 minutes in the dark on ice. Finally, the cells were washed twice with phosphate-buffered saline and fixed in 1% paraformaldehyde (Leiden University Medical Center pharmacy). Immediately after fixation, the cells were acquired on the LSR Fortessa (Fortessa X20 SORP [Special Research Product], Becton, Dickinson and Company) and were analyzed using DIVA software version 8.02 (Becton, Dickinson and Company). The gating strategy was the same as previously described.^25^

Additional high-dimensional single-cell data analysis was performed using hierarchical stochastical neighbor embedding in Cytosplore^26^ after manual gating of the CD3^+^ T cells using Cytobank.

### Stimulation with Autologous Tumor

Tumor-infiltrating lymphocytes were thawed and rested overnight in T-cell medium in the incubator at 37° C (5% CO_2_, 92% relative humidity). The next day, cells were washed and resuspended in Iscove’s modified Dulbecco’s medium with 5% fetal calf serum and IL-2 (25 IU/ml). The cells were seeded in 96-well U- or flat-bottomed plates. The TILs were stimulated with short-term cultured autologous tumor cells. As a positive control, TILs were stimulated with staphylococcal enterotoxin B (Sigma-Aldrich). The cells were tested for reactivity against the autologous tumor by measuring interferon γ (IFN-γ) in the culture supernatant using an enzyme-linked immunosorbent assay for IFN-γ (according to the manufacturer [Sanquin]).^26^ Responses were considered reactive when the mean concentration of IFN-γ–producing TILs cultured with autologous tumor was higher than the mean plus twice the standard deviation of medium control (unstimulated TILs).

### Immunohistochemical Staining

Immunohistochemical staining of BAP1 was performed in all 30 tumors, as previously described.^27^ Tumors were scored as BAP1-positive or BAP1-negative expression based on nuclear staining.

### Chromosome Status

Information on chromosome 3 and 8q on all included UM samples was obtained from the patient’s charts as tests are routinely performed at the Department of Clinical Genetics of the Leiden University Medical Center by single-nucleotide polymorphism analysis.

### RNA Sequence and Cibersort Analysis

RNA library preparation and sequencing of archived snap-frozen tissue of primary UM tumors were performed at Genomescan. Quality control of the RNA sequencing reads as well as adapter clipping, read alignment, and gene expression quantification was carried out using the BIOWDL RNA-Seq pipeline version 1.1.0 (https://doi.org/10.5281/zenod0.3479134). In short, reads were aligned to the human reference genome (hg38 build) using Spliced Transcripts Alignment to a Reference version 2.6.0c,^28^ followed by gene expression quantification by htseq-count version 0.9.1.^29^ For expression quantification, gencode version 30 was used for gene annotation.

Differential expression analysis was performed using the tidybulk R package version 1.2.0 (https://doi.org/10.5281/zenod0.4312265) with default values. In summary, expression values were normalized using the Trimmed Mean of M-values method, and differential expression was calculated with quasilikelihood estimation method in edgeR version 3.32.0.^30^^,^^31^ Normalized counts were extracted for the list of immunologic genes used by Robertson et al,^21^ and the values were plotted in a heatmap using the tidyHeatmap R package (https://doi.org/10.21105/joss.02472). Cell type proportions were inferred from RNA-Seq data using CIBERSORT version 1.04 (https://doi.org/10.1038/nmeth.3337).

### Statistical Analysis

Analyses were performed using SPSS software version 25.0.2 (IBM SPSS Statistics, IBM Corp). Graphs were made using GraphPad Prism version 8.4.2 for Windows (GraphPad Software). Clinical, histopathologic, and genetic parameters were compared between groups using the Pearson chi-square test for categorical prognostic parameters and the Mann–Whitney *U* test for continuous prognostic parameters. A *P* value of < 0.05 was considered statistically significant.

## Results

### Tumor-Infiltrating Lymphocyte Isolation and Tumor-Specific Clinical and Histopathologic Parameters

**Figure 1:**
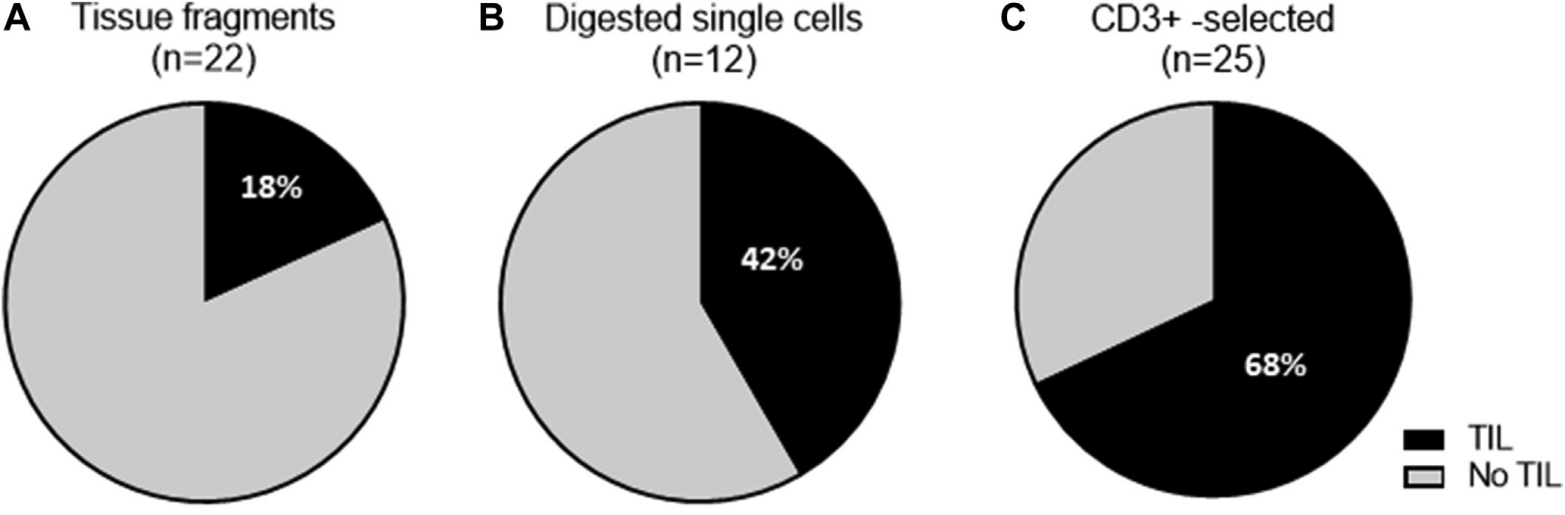
Pie graphs showing a comparison of the success rate of tumor-infiltrating lymphocyte (TIL) expansion in uveal melanoma using 3 different techniques (n = 30 tumors): (**A**) directly from small fragments of fresh tumor tissue (n = 22), with successful TIL expansion in 4 of 22 tumors (18%); (**B**) mononuclear cells obtained by centrifugation of single-cell tissue suspensions after enzymatic digestion, with successful TIL expansion in 5 of 12 tumors (42%); and (**C**) CD3^+^ T cell selection from single-cell preparations using Dynabeads, with successful TIL expansion in 17 of 25 tumors (68%).

Material from 30 primary UM tumors was used to develop a successful protocol to expand TILs. The mean±standard deviation age of the 30 patients at the time of enucleation was 65.2 ± 13.1 years, and 21 patients (74%) were men. T-cell expansion was successful in 19 of the 30 tumor specimens. Success was dependent on the isolation technique: TILs were successfully grown directly from fresh tumor tissue in 4 of 22 tumors (18%), in 5 of 12 tumors (42%) when mononuclear cells were obtained by centrifugation of single-cell tissue suspensions after enzymatic digestion, and in 17 of 25 tumors (68%) after selection of CD3^+^ T cells from single-cell preparations using Dynabeads (Fig 1). Clearly, separation of T cells from the TME was essential to obtain good T-cell expansion. Only the tumors and TILs in the group treated with anti-CD3^+^ Dynabeads were used for the subsequent analyses.

After establishing that T-cell expansion was most successful when T cells were removed from their immunosuppressive context, we considered the possibility that a different genetic background in the tumors impacted our ability to expand TILs, because we previously noted that tumors with monosomy 3 and BAP1 loss contained more T cells.^12^ Therefore, we compared tumor characteristics between the tumors that gave rise to T-cell expansion and those that did not. Focusing on the 25 tumors in which CD3^+^ T cells had been isolated with Dynabeads before T-cell expansion, we compared the clinical, genetic, and histopathologic parameters of the 8 tumors with no T-cell expansion versus the 17 that did give rise to T-cell expansion (Table 1). No differences were observed between the 2 groups with regard to patient age or sex. The tumor’s largest basal diameter was not related to the capacity to expand TILs. However, tumor height was different between the 2 groups: tumors with TIL expansion showed a higher median tumor height compared with tumors without TIL expansion (median, 7.0 mm vs. 4.0 mm, respectively; *P* = 0.02). No differences were found in chromosome status, BAP1 expression, or pigmentation between the 2 groups. Thus, besides tumor height, none of the other studied clinical, histopathologic, or genetic tumor characteristics differed between UM that gave rise to TIL expansion and those that did not.

**Table 1:** Comparison of Clinicohistopathologic and Genetic Features between Primary Uveal Melanoma Tumors (Treated with Anti-CD3 Dynabeads) with and without Successful Tumor-Infiltrating Lymphocyte Expansion and with and without Autologous Tumor-Infiltrating Lymphocyte Reactivity

Clinicohistopathologic Features	Successful Tumor-Infiltrating Lymphocyte Expansion			Autologous Tumor-Infiltrating Lymphocyte Reactivity		
	Without (n = 8)	With (n = 17)	*P* Value∗	Without (n = 9)	With (n = 8)	*P* Value∗
Sex			0.43†			0.49†
Male	6 (38)	10 (63)		6 (60)	4 (40)	
Female	2 (22)	7 (78)		3 (43)	4 (57)	
Age at enucleation (yrs)	72.7 (57.4–78.3)	67.4 (32.2–92.7)	0.18‡	61.9 (32.2–92.7)	68.2 (56.3–84.9)	0.48‡
Largest basal diameter (mm)	10.0 (2–16)	12.5 (9–15)	0.11‡	13 (9–15)	11.5 (10–15)	0.72‡
Height (mm)	4.0 (0–7)	7.0 (3–12)	**0.02**‡	6.5 (3–12)	7 (5–12)	0.44‡
Status			0.27†			0.33†
Alive	7 (30)	16 (70)		8 (50)	8 (50)	
Death resulting from metastases	0	1 (100)		1 (100)	0	
Death resulting from other causes	1 (100)	0		0	0	
Ciliary body involvement			0.32†			0.20†
No	5 (42)	7 (58)		5 (71)	2 (29)	
Yes	3 (23)	10 (77)		4 (40)	6 (60)	
Cell type			0.57†			0.27†
Spindle	1 (50)	1 (50)		0	1 (100)	
Mixed or epithelioid	7 (30)	16 (70)		9 (56)	7 (44)	
Scleral ingrowth			0.74†			0.45†
None or superficial	7 (33)	14 (67)		8 (57)	6 (43)	
Deep, total, or episcleral	1 (25)	3 (75)		1 (33)	2 (67)	
Pigmentation status tumor			0.36†			0.49†
None or low	1 (17)	5 (83)		2 (40)	3 (60)	
Moderate or high	7 (37)	12 (63)		7 (58)	5 (42)	
Break through Bruch’s membrane			0.07†			0.60†
No	4 (67)	2 (33)		1 (50)	1 (50)	
Yes	4 (27)	11 (73)		5 (46)	6 (54)	
Unclear	0	4 (100)		3 (75)	1 (25)	
Metastases			0.31†			0.93†
No	8 (35)	15 (65)		8 (53)	7 (47)	
Yes	0	2 (100)		1 (50)	1 (50)	
Chromosome 3 status			0.89†			0.23†
No monosomy 3	4 (31)	9 (69)		6 (67)	3 (33)	
Monosomy 3	4 (33)	8 (67)		3 (38)	5 (63)	
Chromosome 8q status			0.28†			0.60†
Normal	3 (50)	3 (50)		2 (67)	1 (33)	
Gain or amplification of 8q	5 (26)	14 (74)		7 (50)	7 (50)	
BAP1 protein expression			0.56†			0.70†
Positive	4 (29)	10 (71)		3 (50)	3 (50)	
Negative	4 (40)	6 (60)		6 (60)	4 (40)	

BAP1 = BRCA1-associated protein 1.

Data are presented as no. (%) or median (range). Percentages are rounded and may not total 100.

∗ *P* < 0.05 was considered significant.

† Pearson chi-square test.

‡ Mann–Whitney *U* test.

### Genetic Profiling of the Tumor Environment by RNA Sequencing

**Figure 2:**
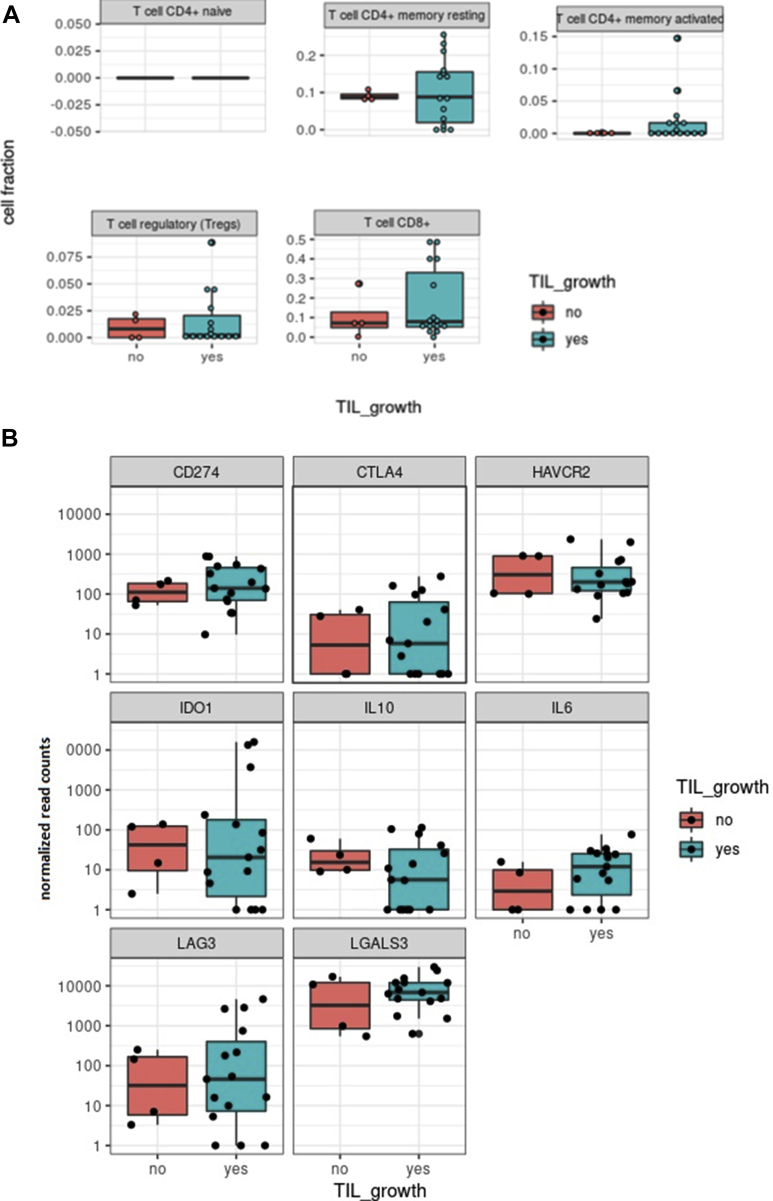
**A**, Graphs showing the relationship between tumor-infiltrating lymphocyte (TIL) expansion and expression of T-cell markers in primary uveal melanoma (UM) tumors determined by RNA-Seq. The expression of different T-cell markers was determined by RNA sequencing in tumors with and without successful TIL expansion after CD3^+^ T-cell selection using Dynabeads (n = 19). The expression of different T-cell markers was further analyzed and plots were made using Cibersort. A comparison between tumor samples without TIL expansion (n = 4, red graphs) and with successful TIL expansion (n = 15, blue graphs) was made. No differences in the expression of T-cell markers were observed between these groups. **B**, Graphs showing the relationship between TIL expansion and expression of immune inhibitory surface markers on primary UM tumors using Cibersort. The expression of inhibitory surface markers was determined by RNA sequencing in tumors with and without successful TIL expansion after CD3^+^ T-cell selection using Dynabeads (n = 19). The expression of inhibitory surface markers was further analyzed and plots were made using Cibersort. A comparison between tumor samples without TIL expansion (n = 4, red graphs) and with successful TIL expansion (n = 15, blue graphs) was made. No differences in the expression of T-cell markers were observed between these groups. CD = cluster of differentiation; CD274 (PD-L1) = programmed death-ligand 1; CTLA4 = cytotoxic T-lymphocyte-associated protein 4; HAVCR2 (TIM-3) = T-cell immunoglobulin and mucin-domain containing 3; IDO1 = indoleamine 2,3-dioxygenase-1; IL10 = interleukin-10; IL6 = interleukin-6; LAG3 = lymphocyte activating 3; LGALS3 = galectin 3.

**Figure 3:**
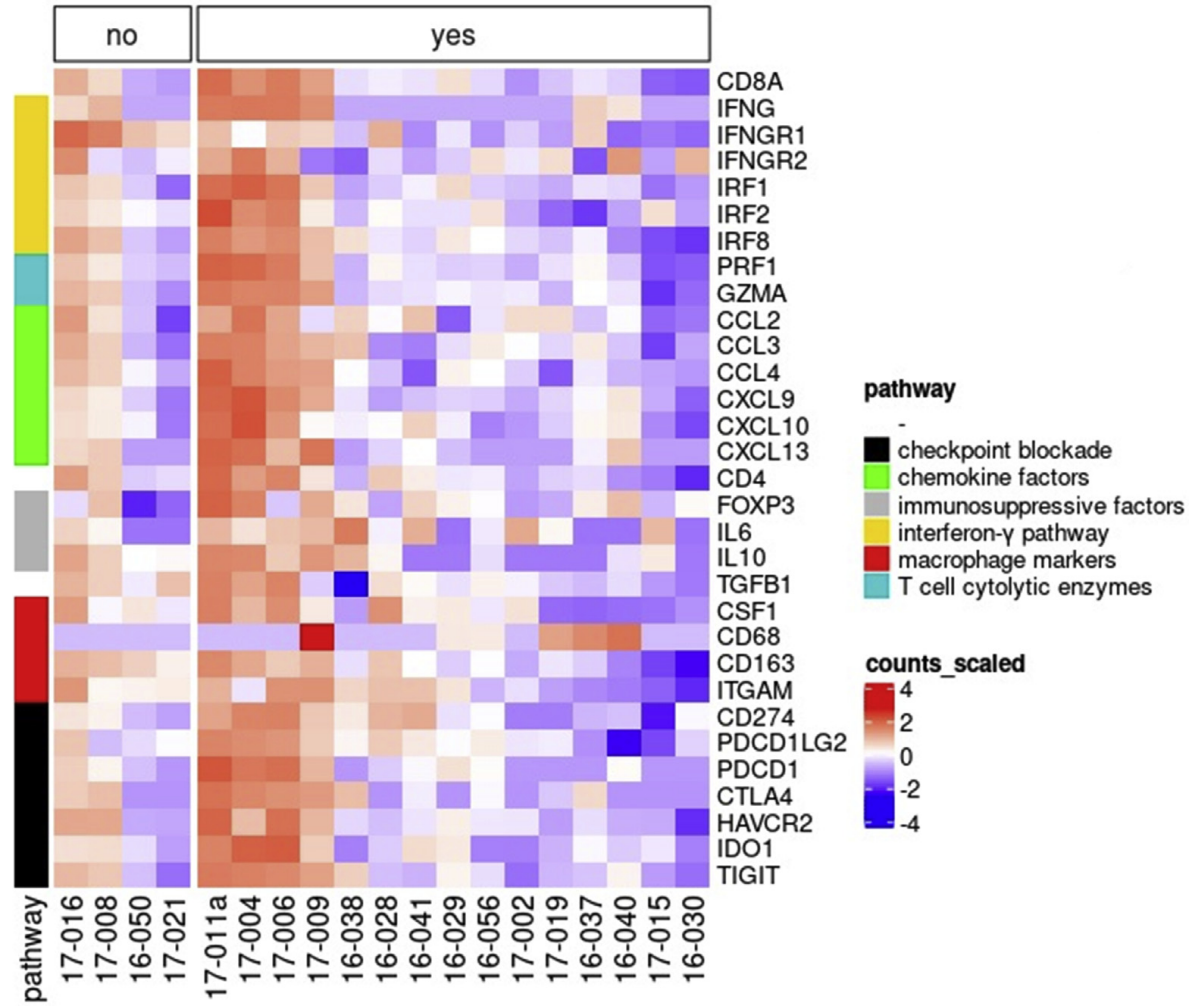
Heatmap showing expression of immunologic markers in primary uveal melanoma with and without successful tumor-infiltrating lymphocyte (TIL) expansion. The expression of immunologic markers was determined by RNA sequencing. Heatmap showing the normalized RNA expression counts of each marker (y-axis) in each of the 19 tumor samples used for CD3^+^ T-cell selection with Dynabeads (x-axis). Highest expression is indicated in red and lowest expression is indicated in blue. Comparison was made in tumors without TIL expansion (n = 4, no) and tumors with TIL expansion (n = 15, yes). No differences were observed when comparing the groups. CCL = C-C motif chemokine ligand; CD = cluster of differentiation; CSF1 = colony stimulating factor 1; CTLA4 = cytotoxic T-lymphocyte-associated protein 4; CXCL = C-X-C motif chemokine ligand; FOXP3 = forkhead box P3; GZMA = granzyme A; HAVCR2 (TIM-3) = T-cell immunoglobulin and mucin-domain containing 3; IDO1 = indoleamine 2,3-dioxygenase-1; IFNG = interferon γ; IFNGR = interferon-γ receptor; IL = interleukin; IRF = interferon regulatory factor; ITGAM = integrin subunit α M; PDCD1 = programmed cell-death protein 1; PDCD1LG2 = programmed cell-death 1 ligand 2; PRF1 = perforin 1; TGFB1 = transforming growth factor β 1; TIGIT = T-cell immunoreceptor with immunoglobulin and ITIM domain.

To determine the presence and abundance of immunosuppressive factors and cells that may cause local immunosuppression, we investigated the distribution of several T-cell subsets and expression of specific myeloid markers and T-cell effector and exhaustion markers in 19 available original tumor tissues (n = 15 expanders after CD3^+^ T-cell selection; n = 4 nonexpanders after CD3^+^ T-cell selection) using RNA sequencing. No differences were observed between the distribution of *CD4*^*+*^-naïve T cells, *CD4*^*+*^ memory T cells*, CD8*^*+*^ T cells, and T regulatory cells in tumors with and without TIL expansion (Figs 2A and 3). Expression of molecules involved in the suppression of T cells, such as *galectin 3*, *programmed death-ligand 1*, indoleamine 2,3-dioxygenase-1, *CTLA4*, *IL-6*, *IL-10*, and *TIM-3*, was highly variable, but no significant differences were found when comparing the groups with and without TIL expansion (Figs 2B and 3). However, a number of molecules were expressed at higher levels in approximately half of all the tested tumors. This high expression illustrates a generally immunosuppressive microenvironment in UM tumors and may explain why the success rate increased from 18% to 68% when T cells were isolated from their TME before expansion.

### Tumor-Infiltrating Lymphocyte Reactivity against the Autologous Tumor

**Figure 4:**
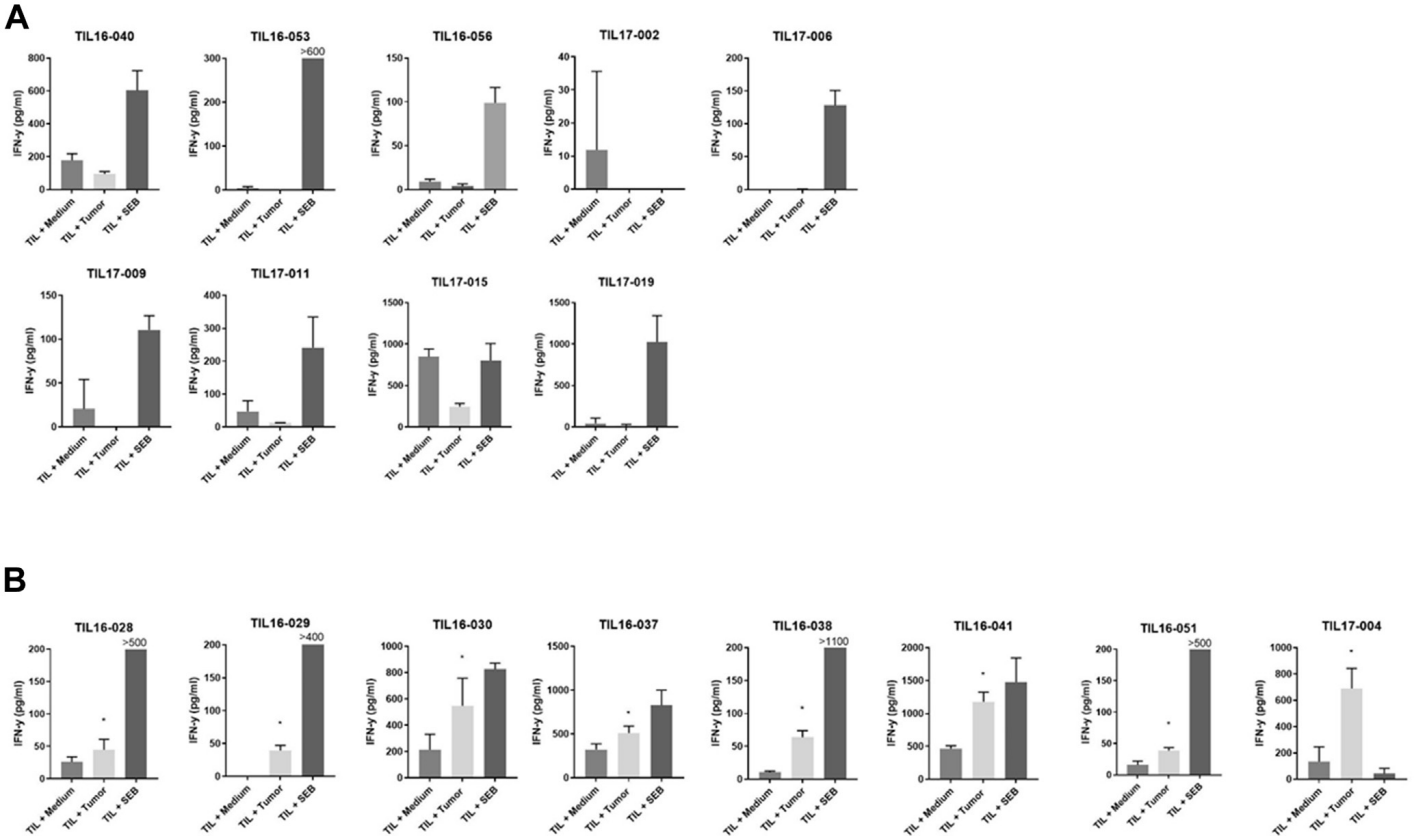
Bar graphs showing tumor-infiltrating lymphocyte (TIL) reactivity against autologous tumor cells. Tumor-infiltrating lymphocytes expanded after CD3^+^ T-cell selection (n = 17) were used to evaluate the TIL reactivity against autologous tumor cells by measuring the interferon γ (IFN-γ) response in the culture supernatant using enzyme-linked immunosorbent assay. Tumor-infiltrating lymphocytes were incubated with medium (medium), with cultured autologous tumor cells (tumor), and with staphylococcal enterotoxin B (SEB) as a positive control. Responses were considered reactive (indicated by the asterisk) when the mean concentration of IFN-γ produced by TILs cultured with autologous tumor was higher than the mean concentration IFN-γ plus twice the standard deviation of medium control (unstimulated TILs). Results of the nonreactive TIL (n = 9) are depicted in (**A**) the upper panels and results of the reactive TIL (n = 8) in (**B**) the lower panels.

**Figure 5:**
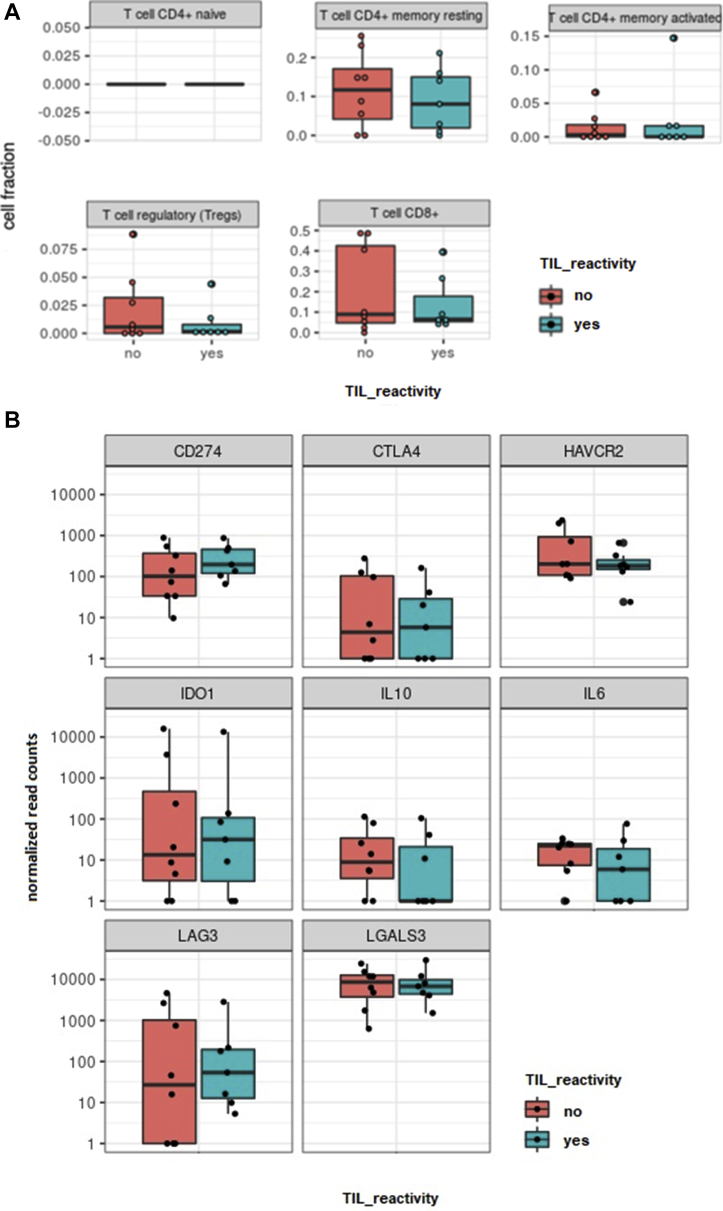
**A**, Graphs showing a comparison of T-cell marker expression in primary uveal melanoma (UM) with and without an autologous T-cell response using Cibersort. The expression of different T-cell markers was determined by RNA sequencing in tumors with successful tumor-infiltrating lymphocyte (TIL) expansion after CD3^+^ T-cell selection using Dynabeads (n = 15). The expression of different T-cell markers was analyzed and plots were made using Cibersort. A comparison was made between tumor samples with TIL that did not (n = 8, red graphs) and did (n = 7, blue graphs) show an autologous tumor response. No differences in the expression of T-cell markers were observed. **B**, Graphs showing a comparison of inhibitory surface marker expression in primary UM with and without an autologous T-cell response. The expression of inhibitory surface markers was determined by RNA sequencing in tumors with successful TIL expansion after CD3^+^ T-cell selection using Dynabeads (n = 15). The expression of inhibitory surface markers was analyzed and plots were made using Cibersort. A comparison was made between tumor samples with TIL that did not (n = 8, red graphs) and did (n = 7, blue graphs) show an autologous tumor response. No differences in the expression of T-cell markers were observed. CD = cluster of differentiation; CD274 (PD-L1) = programmed death-ligand 1; CTLA4 = cytotoxic T-lymphocyte-associated protein 4; HAVCR2 (TIM-3) = T-cell immunoglobulin and mucin-domain containing 3; IDO1 = indoleamine 2,3-dioxygenase-1; IL10 = interleukin-10; IL6 = interleukin-6; LAG3 = lymphocyte activating 3; LGALS3 = galectin 3.

**Figure 6:**
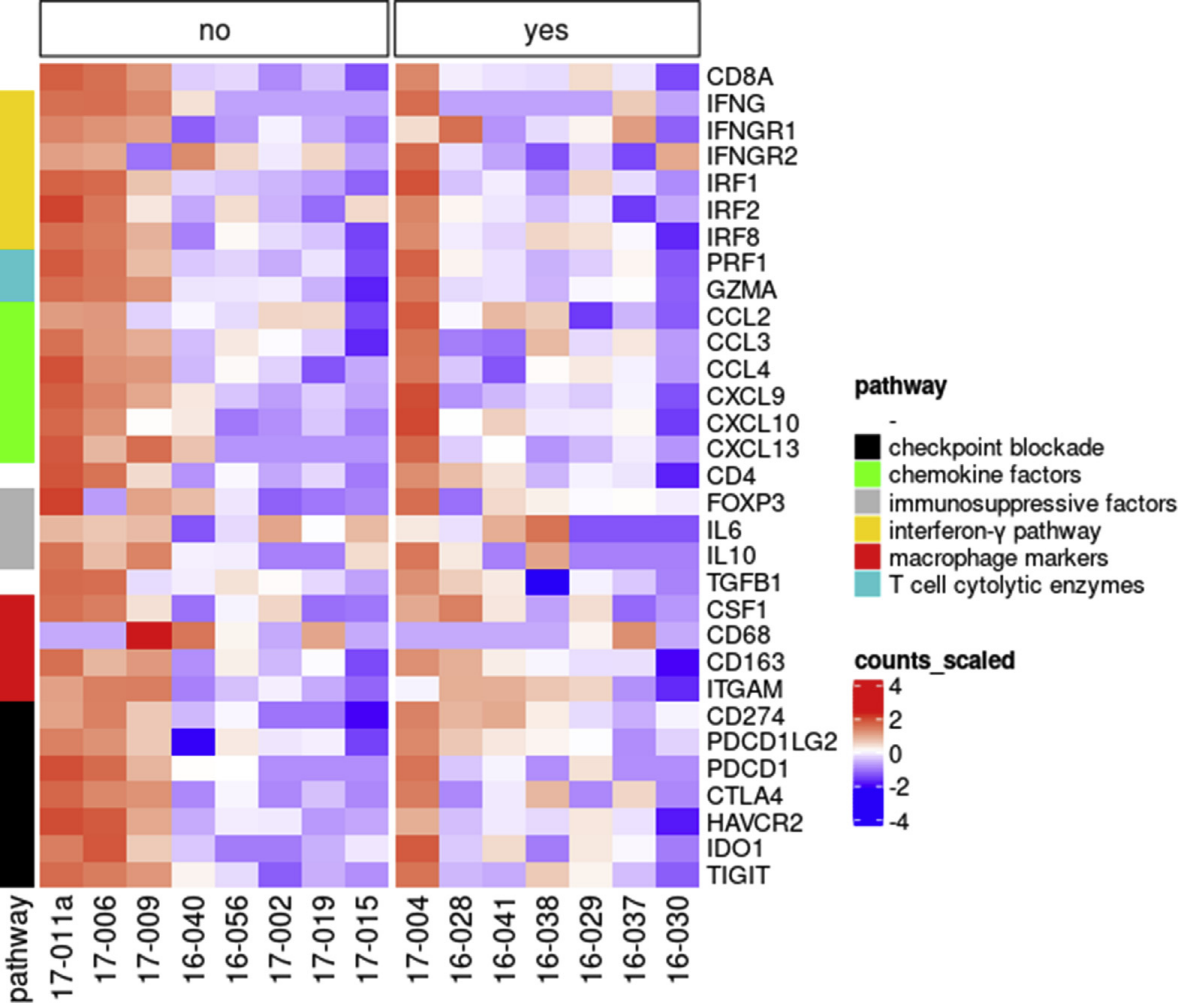
Heatmap showing comparison of immunologic markers in primary uveal melanoma with and without reactive tumor-infiltrating lymphocyte (TIL) cultures using RNA-Seq. The expression of immunologic markers was determined by RNA sequencing. Heatmap shows the normalized RNA expression counts of each marker (y-axis) in each of the 15 tumor samples with successful TIL expansion after CD3^+^ T-cell selection with Dynabeads (x-axis). Highest expression is indicated in red and lowest expression is indicated in blue. Comparison was made in tumors with nonreactive TIL (n = 8, no) and tumors with reactive TIL (n = 7, yes). High expression of immunologic markers was seen in both groups, but independent of TIL culture reactivity. CCL = C-C motif chemokine ligand; CD = cluster of differentiation; CSF1 = colony stimulating factor 1; CTLA4 = cytotoxic T-lymphocyte-associated protein 4; CXCL = C-X-C motif chemokine ligand; FOXP3 = forkhead box P3; GZMA = granzyme A; HAVCR2 (TIM-3) = T-cell immunoglobulin and mucin-domain containing 3; IDO1 = indoleamine 2,3-dioxygenase-1; IFNG = interferon γ; IFNGR = interferon-γ receptor; IL = interleukin; IRF = interferon regulatory factor; ITGAM = integrin subunit α M; PDCD1 = programmed cell-death protein 1; PDCD1LG2 = programmed cell-death 1 ligand 2; PRF1 = perforin 1; TGFB1 = transforming growth factor β 1; TIGIT = T-cell immunoreceptor with immunoglobulin and ITIM domain.

As recently reported by others, tumor-infiltrating T cells do not necessarily recognize tumor antigens.^32^ To assess if the cultured TIL obtained after CD3^+^ T-cell selection comprised tumor-reactive T cells, the 17 TIL cultures were stimulated with autologous UM tumor cells; the detection of IFN-γ in the supernatant was used as measure for TIL responsiveness (Fig 4). The TILs were incubated with medium as the negative control, with cultured autologous tumor cells to test the tumor-reactive TIL response, and with staphylococcal enterotoxin B as the positive control. A TIL response against UM was detected in 8 of 17 TIL cultures. Responsiveness was not related to any clinical, histopathologic, or genetic characteristic of these tumors (Table 1), nor to any specific T-cell type or expression of specific immunosuppressive molecules in the TME (Figs 5A, B and 6), suggesting that the priming and homing of UM-reactive T cells is not overtly influenced by an immune-suppressive TME.

### Tumor-Infiltrating Lymphocyte Phenotyping

To address whether frequencies of CD3^+^CD4^+^ TILs and CD3^+^CD8^+^ TILs and their activation or exhaustion state in vivo affect their autologous tumor-cell reactivity, we performed extensive phenotyping by flow cytometry of 15 of 17 bulk TIL cultures after CD3^+^ selection with Dynabeads; 2 bulk cultures could not be analyzed because of lack of sufficient cells. The TILs were stained for CD3, CD4, and CD8 lineage markers and for several inhibitory surface makers, such as CD56, PD-1, TIM-3, CTLA4, CD94, and NKG2A.

When analyzing the expanded viable cells, the median percentage of CD3^+^ cells was 99% (range, 94%–100%). Among this CD3^+^ TIL population, the median percentage of CD4^+^ cells was 25% (range, 0%–91%) and 39% of cells CD8^+^ (range, 6%–84%). We assessed the CTLA4, PD-1, and TIM-3 expression of these CD4^+^ and CD8^+^ TILs. Among the CD8^+^ TILs, the median percentage of CD8^+^PD-1^+^ cells was 22% (range, 1.5%–74%), that of CD8^+^TIM-3^+^ cells was 48% (range, 9.5%–89%), and that of CD8^+^CTLA4^+^ cells was 0.7% (range, 0.1%–10%). Among the CD4^+^ TILs, the median percentage of CD4^+^PD-1^+^ cells was 40% (range, 3.8%–88%), that of CD4^+^TIM-3^+^ cells was 58% (range, 10%–79%), and that of CD4^+^CTLA4^+^ cells was 1.7% (range, 0.1%–30%). The median percentage of double-positive PD-1^+^TIM-3^+^ cells among the CD8^+^ TILs was 16% (range, 0.2%–29%) and that among the CD4^+^ TILs was 26% (range, 2.4%–53%). A comparison of the fractions of T cells expressing coinhibitory molecules in the UM TIL cultures with or without a response to autologous tumor cells showed no overt differences for CD8^+^ TILs or CD4^+^ TILs (Supplemental Table 1).

**Figure 7:**
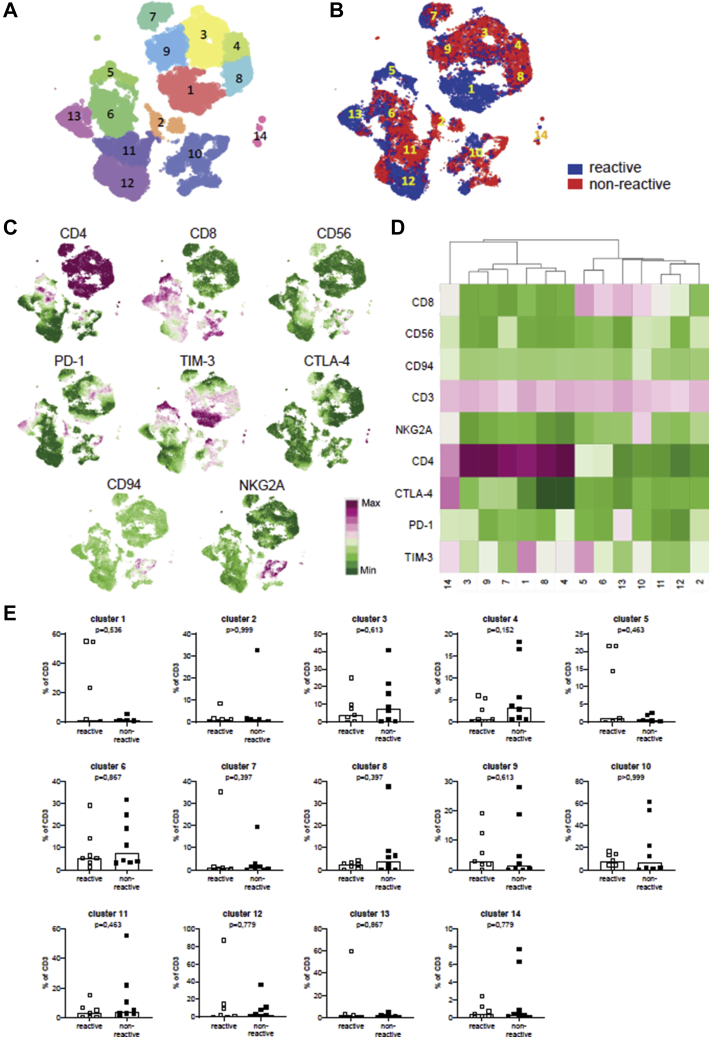
Comparison of inhibitory surface marker expression on (non)reactive tumor-infiltrating lymphocyte (TIL) cultures as determined by flow cytometry. The surface expression of inhibitory markers was determined in TIL cultures obtained after CD3^+^ T-cell selection that were reactive (n = 7) or nonreactive (n = 8) to autologous tumor cells by multicolor flow cytometry using a panel of fluorescently labeled antibodies (see Supplemental Table 1). The expression of the inhibitory markers was further analyzed after gating of the CD3^+^ cells within the live cell populations using Cytosplore software. **A**, Hierarchical stochastic neighbor embedding plotting of data from all TIL cultures, which resulted in definition of 14 different cluster points indicated by the numbers and the different colors. **B**, Distribution of the TIL cultures that were reactive (blue) or nonreactive (red) over the clusters showing their slightly altered cluster geography. **C**, Density plots for each individual marker indicating their cluster geography. Colors represent arcsin150-transformed marker expression, where highest expression is indicted in purple and lowest expression is indicated in green. **D**, Heatmap showing the ^10^log mean fluorescence intensity of each marker (y-axis) in each of the 14 clusters (x-axis). Highest expression is indicated in purple and lowest expression is indicated in green. **E**, Graphs showing a comparison of the frequencies of the 14 clusters of cells as percentage of CD3+ T cells within the analyzed reactive and nonreactive TIL cultures. No significant differences were observed between both groups of TIL cultures (Mann-Whitney *U* test). CD = cluster of differentiation; PD-1 = programmed cell-death protein 1; TIM-3 = T-cell immunoglobulin and mucin-domain containing*-*3; CTLA-4 = cytotoxic T-lymphocyte-associated protein 4.

For subsequent hierarchical stochastical neighbor embedding analysis, CD3^+^ cells were gated and revealed 14 different subpopulations with a distinguishable marker expression profile defined by the cluster points (Fig 7A, C, D). Although the geographical distribution of the reactive (n = 7) and nonreactive (n = 8) TILs over the clusters was slightly different (Fig 7B), no significant differences were observed in cluster frequencies between both groups of TIL cultures (Fig 7E). Although TIM-3–positive clusters 1 and 5 seemed more abundant (Fig 7B) in the tumor-reactive TIL group, no statistical difference was observed when compared with nonreactive TILs (*P* = 0.54 and *P* = 0.46, respectively; Fig 7E).

## Discussion

This study showed that it is possible to culture TILs from freshly enucleated UM and that expansion of TILs is most successful when cells are first separated from their immunosuppressive environment using CD3^+^ T cells with magnetic labeled beads. In approximately half of all tumors, the cultured TILs were able to recognize their corresponding autologous tumor cells. The presence of UM-reactive T cells among TILs was not related to any clinical, histologic, genetic, or immunologic tumor characteristic. However, in approximately half of the UM tumors, the TME overexpressed a number of T-cell suppressive factors, which may explain why it was essential to separate T cells from their tumor environment to successfully achieve TIL expansion. Another factor associated was tumor height; thicker tumors more often gave rise to successful outgrowth of TILs. However, no differences in T-cell counts were observed when comparing the RNA-Seq of the tumors with and without successful TIL expansion in the group with Dynabeads. This indicates that tumors with successful TIL expansion did not necessarily contain more T cells and that the amount of T cells present, and the numbers successfully expanded, were not related to each other. Instead, successful TIL expansion could be related to the viability and functionality of these TILs and the tumor-free environment in which they were cultured.

Our observations are in line with the work of Ksander et al,^23^ who showed that it was possible to grow TILs from primary UM. They obtained TILs from single-cell suspensions derived from UM tissues from 6 patients by adding 1.000 IU/ml recombinant human IL-2, similar to our first protocol. The obtained TILs were able to lyse autologous tumor cells. Interestingly, Verbik et al^22^ showed in another study that T cells successfully expanded in the presence of low concentrations of UM cells, but expansion significantly decreased when more UM cells were added, emphasizing that the presence of UM tumor cells hampers TIL expansion. Also in our study, a number of suppressive factors were identified that could be produced by UM cells (e.g., IL-6, galectin 3) and that may explain why we obtained the highest TIL expansion by separating T cells from their tumor environment. This is supported by our previous finding that galectin 3 hampers tumor-reactive T cells in a mixed lymphocyte–tumor culture.^26^

Another possible UM-related factor that has been mentioned to play a role is the presence of melanin. A study by Rothermel et al^33^ showed that the melanin content differs between metastatic UM and cutaneous metastatic cells, with UM metastatic cells containing higher amounts of melanin. The TILs derived from hypopigmented metastatic UM cells were highly reactive against autologous tumor compared with hyperpigmented metastatic UM cells. No analysis was performed on primary UM. However, in our study, we did not detect a difference in TIL expansion or TIL reactivity between pigmented and nonpigmented primary UM.

Other features that could hamper TIL expansion include the exhaustion status of the T cells in the TME, reflected by the surface expression of coinhibitory markers such as CTLA4, PD-1, and TIM-3.^34^^,^^35^ These markers are known to contribute to the inhibition of T-cell proliferation and activation. It is thought that CTLA4 plays a role in this process in early T-cell development and that PD-1 regulates this inhibition in the later effector phase by binding to its ligands programmed death-ligand 1 or PD-L2.^36^ Furthermore, TIM-3 has an important role in T-cell exhaustion. Blocking the TIM-3 pathway could increase tumor immunity and the production of IFN-γ.^35^ However, in our study, no significant differences were observed in the fractions of molecule expression in primary UM and expanded TILs. Metastatic UM reacts poorly on checkpoint inhibitors (such as ipilimumab and nivolumab),^37^ possibly because T cells may be exhausted, but other immunosuppressive elements, such as galectin 3, may have a more dominant negative influence overruling the effect of checkpoint inhibition.

Despite the successful treatment methods for primary UM, metastatic UM still occurs in half of the patients after enucleation.^1^^,^^37^ Several risk factors increase the probability of metastasis developing in these patients, such as tumor characteristics and chromosomal aberrations.^1^^,^^3^^,^^4^ It is for these patients that adjuvant therapy might reduce the risk of metastatic disease developing. Because immune checkpoint inhibitors show little effect on metastatic UM, adoptive TIL therapy might be a good alternative to treat primary UM patients with high risk of developing metastasis. In a phase 2 trial, adoptive TIL therapy was applied to treat patients with metastatic UM. Thirty-five percent of these patients achieved tumor regression. This included patients in whom treatment with anti-PD-1 and anti-CTLA4 immunotherapy failed. An important explanation for the discrepancy in success for both immunotherapy approaches could be the fact that patients receiving TIL therapy received a chemotherapy-conditioning regimen before TIL infusion that may have eliminated immunosuppressive elements, which is not the case for immune checkpoint inhibitors.^10^

As with other therapeutic methods, adoptive TIL therapy is associated with certain risks. In the reported study of TIL therapy in patients with UM, all patients experienced transient grade 3 or more hematologic toxicity (lymphopenia, neutropenia, and thrombocytopenia) that occurred because of the prior lymphodepleting chemotherapy. Low-grade and resolved infections were documented, and 1 patient died after infection leading to sepsis with multiorgan failure.^10^ Patients receiving this type of therapy more often experience toxicity during chemotherapy before TIL therapy and subsequent IL-2 infusion after TIL administration.^9^^,^^38^ Therefore, a thorough analysis of the patient’s physical condition, with the risks and benefits of the therapy, should be taken into account when considering treating UM patients with high risk of developing metastatic disease with this type of therapy. In this respect, patients may benefit from an alternative approach of TIL therapy after a less intensive preconditioning regimen not associated with such adverse events.^39^

This study showed that TILs from primary UM tumors can be expanded when T cells are isolated from their immunosuppressive TME and that in at least half of all tumors, these TILs are capable of recognizing their autologous tumor. These data suggest that UM-reactive T cells may also be strongly suppressed in vivo, most likely by factors other than immune checkpoints and related to UM cells themselves. Adoptive TIL therapy could be considered as an adjuvant therapy in primary setting for patients with a high risk of developing metastatic disease.
