# Supplementary material for: Tumor-Infiltrating T Cells Can Be Expanded Successfully from Primary Uveal Melanoma after Separation from Their Tumor Environment
Source: Ophthalmol Sci. 2022 Mar 1;2(2):100132. doi: 10.1016/j.xops.2022.100132 (PMC9560540; doi:10.1016/j.xops.2022.100132)
Supplement: Supplemental Table 1 [file mmc1.docx]

**Supplementary Table 1. TIL phenotyping with different T cell- and inhibitory surface markers.**

|  | | | | | **CD8^+^** | | | | | | | **CD4^+^** | | | | | | |
| --- | --- | --- | --- | --- | --- | --- | --- | --- | --- | --- | --- | --- | --- | --- | --- | --- | --- | --- |
| **TIL*** | **TIL reactivity** | **%CD3^+^** | **%CD4^+^** | **%CD8^+^** | **%PD-1^+^** | **%TIM-3^+^** | **%CTLA4^+^** | **%PD-1^+^ TIM-3^+^** | **%CD94^+^** | **%CD56^+^** | **%NKG2a^+^** | **%PD-1^+^** | **%TIM-3^+^** | **%CTLA4^+^** | **%PD-1^+^ TIM-3^+^** | **%CD94^+^** | **%CD56^+^** | **%NKG2a^+^** |
| 16-028 | yes | 94 | 40 | 43 | 42 | 41 | 0.4 | 18 | 6 | 16 | 12 | 40 | 60 | 1.7 | 24 | 0.4 | 4.1 | 1.7 |
| 16-029 | yes | 98 | 2.9 | 84 | 74 | 28 | 0.6 | 16 | 4.4 | 13 | 8 | 3.8 | 53 | 30 | 2.4 | 0.9 | 3.2 | 1.4 |
| 16-037 | yes | 100 | 91 | 6 | 33 | 61 | 0.4 | 27 | 9.9 | 35 | 62 | 39 | 56 | 1.6 | 32 | 1.3 | 38 | 2.1 |
| 16-038 | yes | 98 | 70 | 26 | 24 | 71 | 0.6 | 19 | 6.1 | 6.1 | 4.6 | 9.1 | 66 | 1.1 | 7.4 | 0.4 | 1.6 | 0.4 |
| 16-041 | yes | 100 | 60 | 27 | 14 | 61 | 2.7 | 12 | 40 | 28 | 34 | 31 | 68 | 2.4 | 27 | 4.3 | 1.2 | 0.9 |
| 16-051 | yes | 99 | 0 | 52 | 2.7 | 17 | 6.9 | 0.3 | 5.2 | 3.3 | 6.2 | 88 | 15 | 3.4 | 15 | 12 | 3.4 | 0 |
| 17-004 | yes | 99 | 16 | 73 | 10 | 42 | 0.8 | 4.8 | 6.7 | 9.8 | 12 | 59 | 52 | 0.5 | 31 | 0.9 | 1.6 | 1.4 |
| 16-040 | no | 100 | 34 | 50 | 32 | 78 | 10 | 28 | 27 | 44 | 30 | 35 | 79 | 2.5 | 32 | 5.6 | 18 | 5.2 |
| 16-053 | no | 100 | 84 | 12 | 30 | 54 | 0.3 | 20 | 0.8 | 44 | 1.7 | 32 | 62 | 0.9 | 23 | 1.3 | 26 | 0.9 |
| 16-056 | no | 100 | 70 | 25 | 19 | 16 | 3.3 | 5 | 4.2 | 46 | 14 | 59 | 29 | 2.2 | 22 | 1.4 | 2 | 1.3 |
| 17-002 | no | 99 | 1.8 | 25 | 18 | 58 | 4.2 | 15 | 18 | 60 | 72 | 57 | 72 | 5.9 | 53 | 26 | 34 | 68 |
| 17-006 | no | 100 | 14 | 36 | 1.5 | 9.5 | 0.1 | 0.2 | 12 | 16 | 6 | 18 | 10 | 0.1 | 2.8 | 0.5 | 5.1 | 0.5 |
| 17-009 | no | 99 | 1.4 | 67 | 15 | 29 | 0.5 | 7.1 | 7.8 | 61 | 11 | 76 | 49 | 0.7 | 44 | 2.7 | 5.5 | 1.5 |
| 17-011 | no | 99 | 1.4 | 41 | 37 | 89 | 3.2 | 29 | 41 | 61 | 41 | 77 | 63 | 0.3 | 47 | 6.5 | 11 | 11 |

*%CD3****^+^****: percentage CD3****^+^****of all viable cells*

*%CD4****^+^*** *and %CD8****^+^****: percentage of CD4****^+^*** *and CD8****^+^*** *within CD3****^+^*** *cells*

*%CD8****^+^****PD1****^+^****, %CD8****^+^****TIM-3****^+^****, %CD8****^+^****CTLA4****^+^****, %CD8^+^PD-1^+^TIM-3^+^, %CD8****^+^****CD94^+^, %CD8****^+^****CD56^+^ and %CD8****^+^****NKG2a^+^: percentage of PD-1****^+^****, TIM-3^+^, CTLA4****^+^,*** *PD1^+^TIM-3^+^, CD94^+^, CD56^+^ and NKG2a^+^ cells within the CD8****^+^*** *cell population*

*%CD4****^+^****PD1****^+^****, %CD4****^+^****TIM-3****^+^****, %CD4****^+^****CTLA4****^+^****, %CD4^+^PD-1^+^TIM-3^+^, %CD4****^+^****CD94^+^, %CD4****^+^****CD56^+^ and %CD4****^+^****NKG2a^+^ : percentage of PD-1****^+^****, TIM-3^+^, CTLA4****^+^,*** *PD1^+^TIM-3^+^, CD94^+^, CD56^+^ and NKG2a^+^ cells within the CD4****^+^*** *cell population*

** No FACS analysis could be obtained from two tumors due to lack of cells (tumor-numbers not shown).*

*Percentages were obtained by DIVA software.
Percentages ≥ 10% were rounded.
Abbreviations: PD-1, Programmed cell death protein 1; TIM-3, T cell immunoglobulin and mucin-domain containing-3; CTLA4, Cytotoxic T-lymphocyte-associated protein 4*

*Percentages ≥ 10% were rounded*
